# Missed opportunities: general practitioner identification of their patients’ smoking status

**DOI:** 10.1186/s12875-015-0228-7

**Published:** 2015-02-04

**Authors:** Jamie Bryant, Mariko Carey, Rob Sanson-Fisher, Elise Mansfield, Tim Regan, Alessandra Bisquera

**Affiliations:** Health Behaviour Research Group, Priority Research Centre for Health Behaviour, University of Newcastle & Hunter Medical Research Institute, HMRI Building, Callaghan, NSW Australia; Clinical Research Design IT and Statistical Support Unit, Hunter Medical Research Institute, University of Newcastle, HMRI Building, Callaghan, NSW 2308 Australia

**Keywords:** General practice, Detection, Smoking, Smoking cessation

## Abstract

**Background:**

In order to provide smoking cessation support to their patients in line with clinical practice guidelines, general practitioners must first ascertain whether their patients’ use tobacco. This study examined (i) the sensitivity, specificity, positive predictive value and negative predictive value of general practitioner detection of smoking, and (ii) the general practitioner and patient characteristics associated with detection of tobacco use.

**Methods:**

Eligible patients completed a touchscreen computer survey while waiting for an appointment with their general practitioner. Patients self-reported demographic characteristics, medical history, and current smoking status. Following the patient’s consultation, their general practitioner was asked to indicate whether the patient was a current smoker (yes/no/unsure/not applicable). Smoking prevalence, sensitivity, specificity, positive predictive value and negative predictive values (with 95% confidence intervals) were calculated using patient self-report of smoking status as the gold standard. Generalised estimating equations were used to examine the general practitioner and patient characteristics associated with detection of tobacco use.

**Results:**

Fifty-one general practitioners and 1,573 patients in twelve general practices participated. Patient self-report of smoking was 11.3% compared to general practitioner estimated prevalence of 9.5%. Sensitivity of general practitioner assessment was 66% [95% CI 59–73] while specificity was 98% [95% CI 97–98]. Positive predictive value was 78% [95% CI 71–85] and negative predictive value was 96% [95% CI 95–97]. No general practitioner factors were associated with detection of smoking. Patients with a higher level of education or who responded ‘Other’ were less likely to be detected as smokers than patients who had completed a high school or below level of education.

**Conclusion:**

Despite the important role general practitioners play in providing smoking cessation advice and support, a substantial proportion of general practitioners do not know their patient’s smoking status. This represents a significant missed opportunity in the provision of preventive healthcare. Electronic waiting room assessments may assist general practitioners in improving the identification of smokers.

## Background

### Burden of tobacco use

Tobacco use is the leading cause of premature mortality and preventable morbidity worldwide, with an estimated global prevalence of 25% [1]. In Australia, smoking rates have steadily declined from an estimated 72% among men and 26% among women in 1945 [2] to 15% overall in 2010 [3]. Tobacco use is a risk factor in the development of a wide range of diseases, including cardiovascular disease, respiratory diseases and up to 90% of all lung cancers [4]. In 2004–2005 smoking caused more than 15,000 deaths in Australia, primarily from cancer and cardiovascular disease, and resulted in more than 750,000 hospital bed days and $669 million dollars in healthcare costs to the hospital system alone [5].

### General practitioner intervention for smoking

In 2009–2010, 83% of the Australian population attend a consultation with a general practitioner [6]. General practitioners are therefore in a unique position to provide advice and support for smoking cessation. There is strong evidence that the majority of general practitioners view the provision of smoking cessation advice as an important part of their role [7,8]. There is also strong evidence for the effectiveness of physician intervention. A Cochrane review of 42 trials found that brief advice from a physician can improve quit rates 1-3% above an unassisted quit rate of 2-3% [9], which is significant in terms of public health impact. However, before general practitioners can provide intervention for smoking cessation, they must first ascertain the smoking status of their patient.

### Sub-optimal detection of smoking in general practice

Despite the known public health benefit of general practitioner intervention for smoking, several studies have demonstrated that detection of smoking by general practitioners during routine clinical encounters is low. In a seminal Australian study comparing general practitioner knowledge of patient smoking status to patient self-report, Dickinson and colleagues found the sensitivity of general practitioner detection of smoking was 56%, meaning that 44% of smokers were not identified as such by their doctor [10]. In a follow-up study conducted six years later, Heywood and colleagues found 34% of smokers were not detected by their general practitioner [11]. Given general practitioners in both studies were aware that their knowledge of patient smoking status would be tested, it is reasonable to assume that detection rates in routine practice were lower. Variable rates of ascertainment of smoking status have also been identified in primary care in the United Kingdom, with complete and accurate detection of smoking status varying from 42.4% to 100% across 24 general practices [12]. In the face of declining smoking prevalence rates in Australia, coupled with increased focus on the provision of preventive healthcare in general practice, it is timely to re-examine current rates of detection of tobacco use in general practice in order to quantify current gaps in the provision of best-evidence practice.

Understanding the factors associated with detection of smoking in general practice is also crucial to improving correct ascertainment of smoking status and therefore the provision of appropriate smoking cessation interventions. While numerous studies have examined the practitioner and patient factors associated with provision or non-provision of smoking cessation advice and support [13,14], few have examined the influence of a range of individual patient and general practitioner factors on *detection*.

### Aims

This study aimed to examine:

1. The accuracy (sensitivity, specificity, positive predictive value and negative predictive value) of general practitioner identification of smoking status compared to patient self-report;
2. The general practitioner and patient characteristics associated with detection of smoking.

## Methods

This cross-sectional study examined a range of health risk behaviours including smoking, alcohol consumption, obesity or being overweight, and cancer screening amongst patients attending general practices in Australia. Only items related to smoking will be reported here. Methodology has been described in detail elsewhere [15-17].

Forty-eight general practices in Melbourne, Sydney and Newcastle, Australia, were approached from a random list generated using the Medical Directory Australia and an online telephone directory. A recruitment package was mailed to identified practices and two follow-up telephone calls made to determine interest in participating. General practices where at least two full time general practitioners agreed to participate were considered eligible.

Patients were approached to participate in the study in the waiting room of consenting general practices by a trained research assistant. Patients were eligible to participate if they were aged 18 years or over, could provide informed consent, and could complete a survey presented on a touchscreen computer. All patients provided informed consent before completing the survey before their appointment with the general practitioner.

General practitioners were asked to self-report demographic and practice information including: age; sex; number of years worked in general practice; and number of sessions per week. To determine accuracy of detection, general practitioners were asked to indicate whether each patient was a current smoker with response options yes, no or unsure. General practitioners could refer to patient medical records when providing their response.

Patients were asked to self-report demographic information including: age; gender; highest level of education; whether they held private health insurance; whether they held a health care concession card; the number of times they had visited their general practitioner in the previous 12 months; and whether they had visited this general practitioner previously. Patients were also asked to report if they had ever been told by a doctor or nurse that they had: high blood pressure; high cholesterol; type 2 diabetes; and/or heart problems (yes/no). Patients were asked to indicate their height in feet and inches or centimetres and weight in stones or kilograms for calculation of body mass index (BMI), with a BMI ≥ 25 kg/m^2^ classified as overweight or obese. Patients were asked to report their frequency of alcohol consumption, with greater than two drinks per day two or more times per week OR four or more drinks on a single occasion classified as risky alcohol consumption. A single item from the New South Wales Health Survey was used to determine smoking status [18]. Participants were asked “Which of the following best describes your smoking status? This includes cigarettes, cigars and pipes” with response options: I smoke daily; I smoke occasionally; I don’t smoke now but I used to; I’ve tried it a few times but never smoked regularly; or I’ve never smoked.

The study received ethical approval from the University of Newcastle Human Research Ethics Committee (Approval no: H-2009-0341) and was ratified by the University of New South Wales Human Research Ethics Committee (HREC09393/UN H-2009-0341) and Monash University Human Research Ethics Committee (2009001860).

### Statistical analysis

The prevalence of smoking, as well as general practitioner and patient characteristics were calculated with 95% confidence intervals (CIs). Participants were classified as smokers if they self-reported daily or occasional smoking; all other participants were classified as non-smokers. General practitioner responses were grouped into two categories: ‘smokers’ if the general practitioner assessed current smoking status as yes, and ‘non-smoker’ if they assessed current smoking status as no. General practitioner responses of ‘unsure’ and ‘not applicable’ were excluded from further analysis. Sensitivity was calculated as the proportion of smokers correctly identified as such by the general practitioner. Specificity was calculated as the proportion of patients correctly identified by the general practitioner as a ‘non-smoker’. Positive predictive value was the proportion of patients classifying themselves as a ‘smoker’ out of the total identified as a smoker by the general practitioner. Negative predictive value was the proportion patients classifying themselves as a non-smoker out of the total identified as a non-smoker by the general practitioner. 95% confidence intervals were calculated for sensitivity, specificity, positive and negative predictive values.

To examine the general practitioner and patient characteristics associated with detection of patients who smoked, generalised estimating equations were undertaken. The analysis was performed only on patients who smoked (n = 177), with the odds of general practitioner detection used as the outcome variable. The following general practitioner variables were initially examined using unadjusted models: gender (male/female); age (25-44/ 45-54/≥55); years in general practice (<5/6-19/≥20); and number of sessions per week (≤5/5.5-10/>10). The following patient variables were initially examined using unadjusted models: gender (male/female); age (18-29/30-44/45-64/≥65); highest level of education (High school or below/Technical certificate, Diploma, University or Other); ethnicity (Aboriginal or Torres Strait Islander of Other/Caucasian); private health insurance (yes/no); health care concession card (yes/no); number of times seen this general practitioner in last 12 months (0/1-2/3-4/5-6/7-8/9-10/more than 10); history of type 2 diabetes (yes/no); history of high blood pressure (yes/no); history of high cholesterol (yes/no); history heart disease (yes/no); overweight or obese (not at risk/at risk); and alcohol consumption (not at risk/at risk).

Due to the low sample size it was necessary to employ variable selection methods. Variables that presented p-values < 0.05 in the unadjusted models were subjected to a backward selection process, whereby variables were removed one at a time from the model based on the highest type 3 p-value. The model was kept if the model fit improved with the Quasi AIC (QIC) decreasing by a minimum of one point. Adjusted odds ratios with 95% CIs and type 3 p-values are reported for the final model. Both unadjusted and adjusted models assumed a binomial distribution and were adjusted for clustering within general practitioners. The results of a forward selection model, in which variables were added one at a time, was also compared to the results of the backward selection model.

## Results

### Sample

Of the 48 general practices approached, 12 agreed to participate. Of the 81 GPs within consenting practices, 53 consented to participate and 51 completed at least one patient checklist and were included in the analysis. One hundred and twenty-five patients reported daily smoking and 52 reported occasional smoking. Demographic and professional characteristics of general practitioners are reported in Table 1. Demographic characteristics and medical history of patients are reported Table 2.

**Table 1:** **General practitioner demographic and professional characteristics (n = 51)**

		**n**	**%**
**Gender**	Male	32	63
	Female	19	37
**Age**	25-44	12	24
	45-54	20	39
	≥55	19	37
**Years in general practice**	<5	21	41
	6-19	8	16
	≥20	22	43
**No of sessions per week**	≤5	27	53
	5.5-10	23	45
	>10	1	2

**Table 2:** **Patient demographic and medical history characteristics (n = 1,573** ^**#**^ **)**

		**n**	**%**
**Gender**	Male	608	39
	Female	965	61
**Age**	18-29	170	11
	30-44	329	21
	45-64	558	35
	≥65	516	33
Highest level of education*	High school or below	612	43
	Technical certificate or Diploma	211	15
	University or Postgraduate	562	39
	Other	47	3.3
**Ethnicity**	Aboriginal or Torres Strait Islander	6	0.4
	Caucasian (i.e. White)	1369	87
	Other	198	13
**Private health insurance**	Yes	916	58
	No	657	42
**Number of times seen this general practitioner in last 12 months^**	0-3	465	31
	4-6	554	37
	7-10	235	16
	More than 10	261	17
**Visited this clinic previously** ^**~**^	Yes	1436	97
	No	48	3
**Health care concession card**	Yes	339	22
	No	1234	78
**History type 2 diabetes**	Yes	134	9
	No	1439	91
**History high blood pressure**	Yes	509	32
	No	1064	68
**History high cholesterol**	Yes	390	25
	No	1183	75
**History heart problems**	Yes	187	12
	No	1386	88
**Overweight or obese** ^**+**^	Not at risk	681	46
	At risk	791	54
**Alcohol consumption** ^**†**^	Not at risk	667	44
	At risk	854	56

^*#*^ Proportions may not add to 100 to due missing data. *n = 141 missing. ^n = 58 missing. ^*~*^ *n- = 89 missing.* ^*+*^ *n = 101 missing.* ^*†*^ *n = 52 missing.*

### Accuracy

General practitioner estimated prevalence of smoking was 9.5%, compared to patient self-report of 11.3%. Sensitivity of general practitioner assessment was 66% [95% CI 59–73] while specificity was 98% [95% CI 97–98]. Positive predictive value was 78% [95% CI 71–85] and negative predictive value was 96% [95% CI 95–97].

### Patient and general practitioner characteristics associated with detection of smoking

The results of the generalized estimating equations examining general practitioner and patient characteristics associated with detection of smoking are reported in Table 3. Both forward and backward selection procedures produced similar results, therefore only the adjusted model resulting from backward selection is presented. There were no general practitioner characteristics associated with detection of smoking. Patients who had attained a higher level of education (i.e., received a technical certificate or diploma or had attended university) or responded ‘Other’ were less likely to be detected as smokers compared to patients who had completed high school or below (OR: 0.52 [95% CI 0.27-0.99]).

**Table 3:** **General practitioner and patient characteristics associated with detection of smoking**

		**Unadjusted OR (95%** **CI)**	**Unadjusted**	**Adjusted OR** ^**#**^ **(95%** **CI)**	**Adjusted**
			**p-value**		**p-value** ^**†**^
**General practitioner characteristics**					
**Gender**	Male	Reference	0.858		
	Female	0.93 (.41-2.11)			
**Age**	25-44	Reference	0.027	Reference	
	45-54	2.85 (1.23-6.60)		2.78 (1.15-6.76)	0.057
	≥55	2.46 (1.08-5.60)		2.38 (0.95-5.96)	
**Years in general practice**	<5		NA		
	6-19				
	≥20				
**Number of sessions per week**	≤5		NA		
	5.5-10				
	>10				
**Patient characteristics**					
**Gender**	Male	Reference	0.920		
	Female	1.03 (0.60-1.78)			
**Age**	18-29	Reference			
	30-44	1.43 (0.73-2.78)			
	45-64	2.57 (1.36-4.87)			
	≥65	2.22 (0.63-7.79)			
**Highest level of education**	High school or below	Reference	0.013	Reference	
	Technical certificate, Diploma, University or Other	0.45 (0.24-0.85)		0.52 (0.27-0.99)	0.046
**Ethnicity**	Aboriginal/Torres Strait Islander or Other	Reference	0.830		
	Caucasian	0.91 (0.38-2.20)			
**Private health insurance**	Yes	Reference	0.036		
	No	2.12 (1.05-4.26)			
**Number of times seen this general practitioner in last 12 months**	0-3	Reference	0.530		
	4-6	1.53 (0.66-3.58)			
	7-10	0.90 (0.38-2.13)			
	More than 10	1.63 (0.69-3.81)			
**Visited this clinic previously**	Yes	Reference	0.717		
	No	1.49 (0.17-12.95)			
**Health care concession card**	Yes	Reference	0.475		
	No	0.78 (0.40-1.53)			
**History type 2 diabetes**	Yes	Reference	0.138		
	No	0.34 (0.08-1.41)			
**History high blood pressure**	Yes	Reference	0.011	Reference	
	No	0.38 (0.18-0.80)		0.48 (0.20-1.10)	0.084
**History high cholesterol**	Yes	Reference	0.076		
	No	0.44 (0.17-1.09)			
**History of heart problems or stroke**	Yes	Reference	0.326		
	No	2.12 (0.47-9.51)			
**Overweight or obese**	Not at risk	Reference	0.070		
	At risk	0.58 (0.32-1.04)			
**Alcohol consumption**	Not at risk	Reference	0.071		
	At risk	0.50 (0.23-1.06)			

*OR = odds ratio. CI = confidence interval.* ^*#*^ *Adjusted for all variables in the final model.*

^*†*^ *p-value from the Wald test. NA = Not available as univariate analysis could not be performed due to low cell counts.*

## Discussion

This cross-sectional study examined the prevalence of smoking and accuracy of detection of smoking status in routine clinical practice by general practitioners. The overall prevalence of smoking was 11.3%, which is lower than the general population smoking prevalence of 18% [19] at the time data was collected. However, this is likely to reflect the high proportion of people over 45 years of age (68%) in the current sample, given that smoking rates demonstrate a steady decline from this age [19].

### Sensitivity and specificity

General practitioner sensitivity in detection of smoking for the current sample was 66%. As identification of smoking status is the necessary first step in the provision of smoking care, this suggests that at least a third of smokers are unlikely to be offered advice or support to quit smoking by their general practitioner. This is a significant proportion and represents an important missed preventive health opportunity, particularly since general practitioner brief advice has been shown to be an effective strategy for encouraging quit attempts [9]. As most smokers require repeated quit attempts in order to successfully quit [20], general practitioner awareness of smoking status provides an opportunity for the provision of ongoing support. Detection of smoking had high specificity (98%). This may reflect the fact that smoking is an uncommon behaviour especially among middle aged and older adults [21], who predominated this sample. This high specificity indicates that general practitioner assessments of a positive smoking status are highly reliable and could be used as the basis for mail-based or within-consultation provision of smoking care advice and support.

### Characteristics associated with detection

Only education level was associated with detection of smoking status, with participants with a higher level of education less likely to be detected than those with a lower level of education. Lower socioeconomic status has been found to be associated with smoking [22], therefore, this finding may reflect general practitioner stereotypes about patient characteristics associated with smoking. Given that smokers of higher socioeconomic status may be more likely adhere to smoking cessation interventions and to quit successfully [22], failure to identify these smokers and provide appropriate care may have a significant overall impact on cessation rates.

### Practice implications

This study showed that electronic health screening in general practice waiting rooms may provide a practical method of improving general practitioner identification of smoking status among their patients. Our data has shown that patients find this method highly acceptable, with the majority agreeing that the touchscreen assessment method is easy and provides sufficient privacy [23]. General practitioners also perceive that this is a practical method of assessment which is not disruptive to the functioning of the clinic [23]. While feedback on patient smoking status was not provided in this study, previous data has indicated that a majority of patients would be happy for this type of information to be kept on file and provided to their doctor to inform provision of clinical care [23]. This may be a particularly useful method of identifying smokers who do not fit the usual stereotype of people who smoke and hence may not be detected as part of routine clinical assessments.

### Study strengths and limitations

This study reported on the accuracy of general practitioner detection of smoking status among a sample of general practice patients. The recruitment of participants from multiple clinics is a strength of the study, however the small number of consenting practices is a limitation [24]. Biochemical assessment of smoking status such as salivary cotinine is the most accurate method for identifying people who smoke [25,26]. However, this method is not feasible for routine implementation in clinical practice, and indeed, would likely undermine the therapeutic relationship. Although self-report data may be subject to social desirability bias, this is the method that is likely to be used in clinical practice for identification of lifestyle risk factors so was considered appropriate for this study.

## Conclusions

Despite the important role general practitioners have in providing smoking cessation advice and support, a substantial proportion of patients’ smoking status is not known by their general practitioner. Smokers with higher education are most likely to remain undetected. Our data indicate that electronic waiting room assessments may assist general practitioners in improving the identification of smokers.

## Abbreviations

BMI: Body mass index
Quasi AIC (QIC): Quasi Akaike's information criterion
CI: Confidence interval
OR: Odds ratio
